# Prepartum body condition score affects milk yield, lipid metabolism, and oxidation status of Holstein cows

**DOI:** 10.5713/ajas.18.0817

**Published:** 2019-04-15

**Authors:** Wei Zhao, Xue Chen, Jun Xiao, Xiao Hui Chen, Xue Feng Zhang, Tao Wang, Yu Guo Zhen, Gui Xin Qin

**Affiliations:** 1 College of Animal Science and Technology, Jilin Agricultural University, Changchun 130118, China; 2 JLAU-Borui Dairy Science and Technology R&D Centre of Jilin Agricultural University, Changchun 130118, China; 3 Key Laboratory of Animal Production, Product Quality and Security, Ministry of Education, Jilin Agricultural University, Changchun 130118, China; 4 Key Laboratory of Animal Nutrition and Feed Science of Jilin Province, Jilin Agricultural University, Changchun 130118, China

**Keywords:** Milk Production, Lipid Metabolism, Oxidative Stress, Transition Period, Body Condition Score Loss

## Abstract

**Objective:**

This study aimed to investigate the effects of prepartum body condition score (BCS) on the milk yield, lipid metabolism, and oxidative status of Holstein cows.

**Methods:**

A total of 112 multiparous Holstein cows were divided into 4 groups according to the BCS at 21 days before calving: medium BCS (3.0 to 3.25, MBCS), high BCS (3.5 to 3.75, HBCS), higher BCS (4.0 to 4.25, HerBCS), and highest BCS (4.5 to 5.0, HestBCS). Blood samples were collected on 21, 14, and 7 days before calving (precalving), on the calving day (calving), and on 7, 14, and 21 days after calving (postcalving). The indices of lipid metabolism and oxidative status were analyzed using bovine-specific enzyme-linked immunosorbent assay kit. Colostrum were taken after calving and analyzed by a refractometer and milk analyzer. The individual milk yield was recorded every 3 days.

**Results:**

The density and levels of immune globulin and lactoprotein of colostrum from Holstein cows in the HestBCS group were the highest (p<0.05). These animals not only had the highest (p<0.05) levels of serum non-esterified fatty acids and beta-hydroxybutyrate, but also had the highest (p<0.05) levels of malondialdehyde, superoxide dismutase, catalase, vitamin A, and vitamin E. In addition, greater (p<0.05) BCS loss was observed in the HestBCS cows.

**Conclusion:**

This study demonstrates that the milk yield, lipid metabolism, and oxidative status of Holstein cows are related to prepartum BCS and BCS loss during the transition period. HestBCS cows are more sensitive to oxidative stress and suffer greater loss of BCS after calving, whereas the MBCS animals had better milk yield performance.

## INTRODUCTION

In the transition period, dairy cows experience oxidative stress and are susceptible to metabolic disorders and immunologic challenges [1,2]. At the time of early lactation, cows go through a period of negative energy balance (NEB) which may lead to oxidative stress and health problems in these animals [3]. Early lactation NEB not only decreases milk yield but also increased concentrations of non-esterified fatty acids (NEFA) and beta-hydroxybutyrate (BHBA) [4,5]. NEFA in the blood is mobilized from adipose tissue while BHBA is an intermediate metabolite of fatty acid oxidation [6]. NEFA and BHBA are considered to be biomarkers of NEB, and oxidative stress during the transition period [7,8]. The body condition score (BCS) is an assessment of body fat and reflects the NEB of Holstein dairy cows [9,10]. It was reported that the BCS of cows were associated with differences in milk production and composition, reproduction, and health [7,11]. Cows with higher BCS at calving had higher concentrations of NEFA [12,13] and BHBA [13] in early lactation compared these with lower BCS. Cows with higher BCS are more sensitive to oxidative stress [14,15]. Oxidative stress is mainly derived from an imbalance between reactive oxygen metabolites and antioxidants and the concentration of some oxidative status indices such as malondialdehyde (MDA) was higher in cows with higher BCS before calving [15].

But the change of BCS during the transition period and their effects on the health and subsequent performance of dairy cows was few systematically studied. Therefore, the relations between the BCS and milk production, lipid metabolism, oxidative status of dairy cows were investigated in the present study. Consequently, this study may be used as reference for the dairy farms to improve the animals’ health status and performance by adjusting prepartum BCS.

## MATERIALS AND METHODS

### Animals and experimental design

The trial was carried out at a commercial dairy farm with about 5,000 lactating cows in Dongying City, Shandong Province, P.R. China. The average milk yield per lactation of the cows was more than 40 kg (305 day). The BCS (5-point scale) was individually recorded 21 days before calving, on the calving day and 21 days after calving [9]. Multiparous Holstein cows (second or third lactation) were selected according to their BCS at 21 days before calving and divided into 4 groups (28 animals per group): medium BCS (3.0 to 3.25, MBCS), high BCS (3.5 to 3.75, HBCS), higher BCS (4.0 to 4.25, HerBCS), and highest BCS (4.5 to 5.0, HestBCS). The cows were given access to total mixed ration and water *ad libitum*. The cows received a far-off diet (1.33 Mcal/kg) from −60 d to −22 d, and a close-up diet (1.50 Mcal/kg) was offered during the last 21 d. From 4 to 6 days postpartum, cows were supplemented with propylene glycol (PG, 300 mL) as a gluconeogenic precursor for both treatment and prevention of ketosis during transition. Cows were fed lactation diet I until 21 d (1.78 Mcal/kg) and lactation diet II (1.83 Mcal/kg) from 22 d to 100 d and milked four times at approximately 6:00, 12:00, 18:00, and 0:00; the individual milk yield was recorded every 3 days. The compositions of the diets are shown in Table 1 [16]. All of the animal experimental procedures were approved by the Institutional Animal Care and Use Committee at Jilin Agricultural University.

### Blood and milk samples

Blood was sampled via the coccygeal vein after morning feeding at days 21, 14, and 7 prepartum (precalving), day 0 (calving), and days 7, 14, and 21 postpartum (after calving). Serum was collected in (1.5 mL) after centrifuging at 2,000×*g* at 4°C for 10 min and stored at −20°C until analytical determination. Colostrum was taken immediately after calving, and the levels of lactoprotein, lactose, solid-not-fat, and ash were analyzed by a milk analyzer (Foss Electric, Hillerød, Denmark) while the density and immune globulin concentration were analyzed by a refractometer (Yucheng To You Optical Instruments Co. Ltd., Shandong, China). The concentrations of NEFA, BHBA, superoxide dismutase (SOD), MDA, catalase (CAT), vitamin A (VA) and vitamin E (VE) in serum were analyzed using commercial bovine specific enzyme-linked immunosorbent assay kits (Shanghai Enzyme-linked Biotechnology Co. Ltd., Shanghai, China) according to the instructions. Reaction is terminated by sulphuric acid. The color change is measured spectrophotometrically at a wavelength of 450 nm (Multiskan FC, Thermo scientific, Waltham, MA, USA) and calculated by comparing the optical density of the samples to the standard curve, respectively.

### Statistical analysis

All of the data are presented as the means±standard error and were subjected to one-way analysis of variance (colostrum composition) and general linear model by SPSS computer software (SPSS Inc., Chicago, IL, USA). The specify model included BCS (MBCS, HBCS, HerBCS, and HestBCS), Time (precalving, calving, postcalving) and their interactions (BCS× time) as fixed effects and cow within the treatment as random effect. Statistical significance is defined when p values are less than 0.05. Pearson correlation coefficients among the detected parameters were also calculated.

## RESULTS

### Colostrum composition, milk yield, and body condition score loss

**Figure 1:**
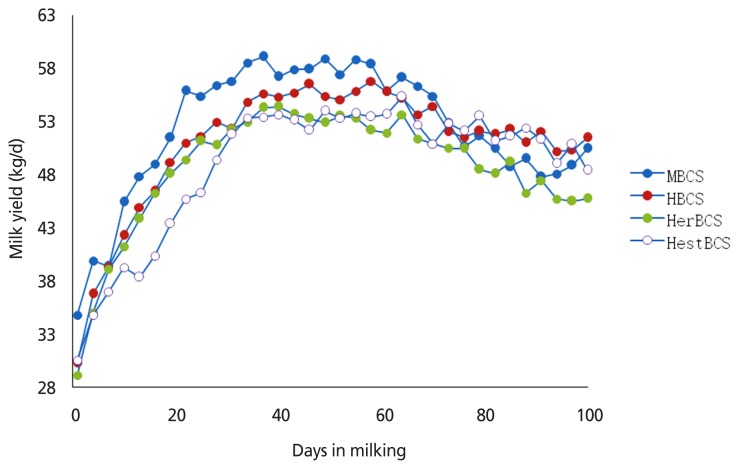
Average milking yield of Holstein cows after calving (0 to 100 days).

**Figure 2:**
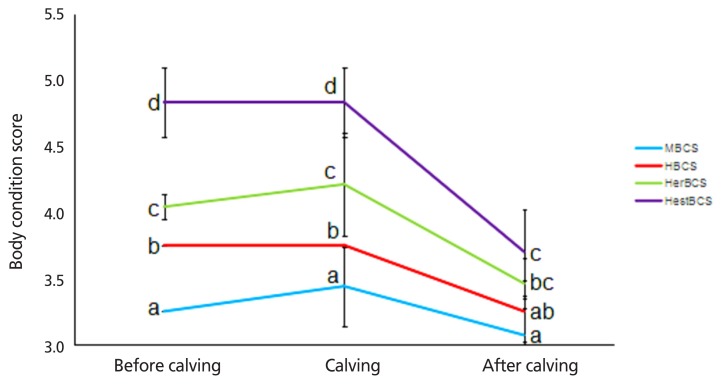
Body condition score of Holstein cows before calving, at calving, and after calving. ^a^^–^^d^ Means in the same row followed by different superscripts differ at p<0.05. The same letter or no letter indicates no significant difference.

The density of colostrum and the levels of immune globulin and lactoprotein of cows in the HestBCS group were higher (p<0.05) than those in the other groups (Table 2). The milk yields of cows in the MBCS, HBCS, HerBCS, and HestBCS groups peaked (≥50 kg/d) at days 19 to 82, 22 to 100, 25 to 73, and 31 to 97, respectively. The average milk yields (0 to 100 days) were 52.35 kg/d, 50.94 kg/d, 48.59 kg/d, and 48.78 kg/d, respectively (Figure 1; Table 2). In addition, the present data showed that the animals in the HestBCS group had a greater BCS loss after calving (Figure 2). The percentages of lost BCS were 23.62% (HestBCS), 14.36% (HerBCS), 13.33% (HBCS), and 5.53% (MBCS) (Figure 2).

### Lipid metabolism and oxidative status

NEFA was affected by both BCS (p<0.01) and time (p<0.01), while BHBA was only affected by BCS (p<0.01). There were no BCS×time effects on NEFA (p = 0.21) or BHBA (p = 0.97) during the transition period (Table 3). The serum levels of NEFA in cows in the HestBCS group on the calving day and after calving were all higher than those observed in other groups (p<0.05). In addition, the concentrations of BHBA were the highest (p<0.05) in HestBCS on precalving, calving, and postcalving days. Table 4 shows that MDA, SOD, and VA were affected by both BCS and time (p<0.05). There were BCS×time effects on SOD and VA during the transition period (p = 0.05). The concentrations of MDA, SOD, CAT, VA, and VE (except during precalving) in HestBCS (4.5 to 5.0) cows were all higher (p<0.05) than those in the other groups. Positive Pearson correlation coefficients were observed for NEFA (r = 0.260), BHBA (r = 0.496), MDA (r = 0.700), SOD (r = 0.606), CAT (r = 0.342), VA (r = 0.245), and VE (r = 0.448) to BCS (Table 5).

## DISCUSSION

In early lactation, dairy cows experience a period of NEB due to the sudden increase in requirements of the mammary glands for milk production [17]. The mobilization of adipose tissue is associated with decreasing body weight (BW), and cows in transition with higher BCS lose more BW [18,19]. Similar results were observed in our study as the cows in the HestBCS group had the greatest BCS loss at 21 d after calving. Loker et al [20] suggested that lower production is associated with greater BCS. In our study, animals in the HestBCS group had a delayed peak milk yield. However, Dechow et al [21] found that fatter cows with more loss of BCS in early lactation have greater milk production. Milk composition is positively associated with BCS, and higher calving BCS means more protein and lactose during early lactation [22,23]. In our research, a higher level of lactoprotein was observed in HestBCS cows.

The NEFA and BHBA are commonly measured as indices of NEB and fat oxidization, and their concentrations during the early postpartum period are associated with BCS [10,19, 24,25]. The NEFA concentration in blood can be a reason for adipose tissue mobilization due to excess acetyl CoA generated from β-oxidation into ketone bodies, this leads to the loss of BCS [26,27]. In the present study, more rapid BCS loss and higher levels of NEFA and BHBA after calving were found in HestBCS cows, which is consistent with the results of Adrien et al [28]. Barletta et al [19] indicated that cows that lost BCS during the transition period had higher concentrations of NEFA and BHBA and more health events during the lactation. However, Mansouryar et al [23] fund that there was no significant differences in concentrations of NEFA and BHBA in high and normal BCS cows in early lactation. A BCS of 3.5 or higher at calving is associated with increased risk for ketosis [15], and a concentration of BHBA ≥1,200 μmol/L is considered as a sign of clinical ketosis [29,30]. However, no cows in the present study were diagnosed as ketotic at 1 week postpartum, as the BHBA concentrations were all lower than 1,200 μmol/L. This may be because choline (STA-CHOL) was added in the close-up diet [31] and PG was orally fed to cows at 4 days postpartum [32,33].

The MDA is the end product of the oxidative destruction of lipids, and its content can indicate the oxidative status of dairy cows [34,35]. In the present study, the highest level of MDA was found in HestBCS cows and MDA were increased on the calving day and decreased after calving. These data demonstrate that cows suffered the most serious oxidative stress on calving day and cows with higher BCS suffered much more serious oxidative stress than the other BCS cows before or after calving. It is probable that a negative association between BCS and dry matter intake in early lactation [36]. To maintain the requirements for milk production, prepartum cows with higher BCS experience more serious NEB [17].

The SOD is considered to be the first defense against pro-oxidants that convert the superoxide (O_2_^−^) to hydrogen peroxide (H_2_O_2_) [37] and Bernabucci et al [38] used SOD in blood to assess the oxidative status of transition dairy cows. In the current study, HestBCS cows had the highest SOD concentrations. And the concentration of SOD was commonly higher on calving day which was similar to the report of Bernabucci et al [15]. CAT is a critical antioxidant enzyme that prevents the accumulation of H_2_O_2_ and is considered to be an index of antioxidative status [39,40]. In the present study, CAT and MDA showed positive correlations, which is consistent with a previous study [35].

The VA and VE are fat-soluble membrane antioxidants that are important for enhancing antioxidant defense systems against oxidative stress [41,42]. Jin et al [43] reported that a diet supplemented with VA increases the concentration of serum SOD and CAT, which is consistent with the results of the current study. The VE supplementation reduces oxidative damage in the livers of heifers [44] and mitigates oxidative stress in crossbred cows [45]. In the present study, an oxidative (NEFA, BHBH, MDA)-antioxidative (SOD, CAT, VA, VE) balance was obviously observed in the cows, which might be the result of homeostatic control.

## CONCLUSION

This study indicates that the milk yield, lipid metabolism, and oxidative status of Holstein cows are related to prepartum BCS and BCS loss during the transition period. Holstein cows with MBCS have better milk yield performance after calving whereas the animals with HestBCS suffer greater BCS loss and higher oxidative stress. Our data may provide some theoretical basis for improving the postpartum health and performance of dairy cows by adjusting prepartum BCS.

## Figures and Tables

**Table 1:** Ingredients (kg/head/d) and chemical composition (% of dry matter) of the experimental diets

Items	Far-off diet (−60 d to −22 d)	Close-up diet (−21 d to calving)	Lactation diet I (calving to 21 d)	Lactation diet II (22 d to 100 d)
Ingredients				
Corn silage	6.00	12.00	14.00	-
Fermented rice straw	7.00	-	-	-
Alfalfa hay	-	-	3.70	4.00
Wet distilled grain solubles	-	3.00	2.00	5.00
Oat grass	6.00	3.80	0.50	-
Steam-flaked corn	-	-	2.50	4.40
Corn	-	1.40	1.40	2.20
Urea	0.04	0.05	0.04	0.07
Soybean	0.70	0.50	1.50	2.30
Spouting corn bran	0.50	-	-	-
Soybean hull	0.50	0.70	0.80	-
Rapeseed meal	1.30	1.00	-	0.50
Extruded soybean	-	-	-	0.50
Premix I1)	0.20	-	-	-
Premix II2)	-	0.80	-	-
Premix III3)	-	-	0.68	0.68
Fat powder	-		0.15	0.30
Rumen bypass protein	-	0.50	1.20	1.20
Diamond V XPC	-	0.025	0.025	0.025
STA-CHOL	-	0.056	0.060	-
Rumen-protected nicotine	-	0.02	0.02	-
KHCO_3_	-	-	0.05	-
Molasses	-	-	0.80	1.00
Wet matter (kg/head/d)	22.24	23.85	29.42	45.18
Dry matter intake (kg/head/d)	13.40	13.10	17.30	24.40
Chemical composition				
Acid detergent fiber	32.92	22.41	18.69	16.64
NEl (Mcal/kg dry matter)4)	1.33	1.50	1.78	1.83
Neutral detergent fiber	49.00	36.60	27.87	26.28
Crude protein	13.00	13.85	16.40	17.36
Starch	13.50	23.30	26.37	30.90
Non-fiber carbohydrates	30.00	37.85	43.81	46.06
Calcium	0.51	1.20	1.09	0.85
Phosphorus	0.39	0.44	0.45	0.45

1) Nutrients provided/kg of additive: vitamin A, 700 kIU; vitamin D_3_, 200 kIU; vitamin E, 8,500 IU; Cu, 650 mg; Zn, 1,656 mg; Mn, 1,335.6 mg; Co, 40 mg; I, 80 mg; Se, 32 mg; CaCO_3_, 122 g; CaHPO_4_·2H_2_O, 34 g; NaCl, 120 g.

2) Nutrients provided/kg of additive: vitamin A, 400 kIU; vitamin D_3_, 100 kIU; vitamin E, 5,000 IU; Cu, 375 mg; Zn, 345 mg; Organic Zn, 225 mg; Mn, 508.8 mg; Co, 6 mg; I, 24 mg; Organic Se, 24 mg; Ca, 143.8 g; P, 13.6 g; Cl, 158.8 g; S, 49.6 g.

3) Nutrients provided/kg of additive: vitamin A, 400 kIU; vitamin D3, 150 kIU; vitamin E, 3,000 IU; Cu, 650 mg; Zn, 2,415 mg; Mn, 1,303.8 mg; Co, 24 mg; I, 60 mg; Se, 24 mg; Ca, 157.7 g; P, 32.3 g; NaCl, 130 g.

4) NEl (Mcal/kg DM) was calculated according to NRC [16].

**Table 2:** Influence of body condition score on the colostrum composition (%) and average milking yield (kg/d, 0 to 100 d) of Holstein cows

Variables	MBCS (3.0 to 3.25)	HBCS (3.5 to 3.75)	HerBCS (4.0 to 4.25)	HestBCS (4.5 to 5.0)
Density (%)	19.49±4.07^a^	20.30±3.19^a^	20.11±4.15^a^	29.39±2.77^b^
Immune globulin (%)	18.77±3.08^a^	18.31±3.22^a^	19.59±3.63^a^	27.34±3.92^b^
Lactoprotein (%	6.06±1.08^a^	5.75±1.38^a^	6.57±0.99^ab^	7.59±0.62^b^
Lactose (%)	8.84±1.14	8.26±1.88	9.21±1.38	9.22±1.61
Solid-not-fat (%)	16.52±2.18	17.12±2.05	18.64±1.52	20.06±1.45
Ash (%)	1.19±0.20	1.12±0.29	1.27±0.18	1.33±0.27
Average milking yield (kg/d)	52.35±7.03	50.94±7.42	48.59±3.83	48.78±5.43

MBCS, medium BCS (3.0 to 3.25); HBCS, high BCS (3.5 to 3.75); HerBCS, higher BCS (4.0 to 4.25); HestBCS, highest BCS (4.5 to 5.0).

a,b Means in the same row followed by different superscripts differ at p<0.05.

**Table 3:** Influence of body condition score on the levels of non-esterified fatty acid (NEFA) and beta-hydroxybutyrate (BHBA) of Holstein cows

Variables		Treatments1)				p-vale		
	
		MBCS (3.0 to 3.25)	HBCS (3.5 to 3.75)	HerBCS (4.0 to 4.25)	HestBCS (4.5 to 5.0)	BCS	Time	BCS×time
NEFA (μmol/L)	Precalving	1,372.50±309.31^B^	1,412.50±311.67^B^	1,535.00±351.96^B^	1,730.00±446.29	<0.01	<0.01	0.21
	Calving	871.14±101.89^a^^A^	774.00±159.33^a^^A^	833.00±126.91^a^^A^	1,369.0±137.25^b^			
	Postcalving	1,510.20±270.87^b^^B^	900.50±147.92^a^^A^	889.75±124.59^a^^A^	1,632.50±153.60^b^			
BHBA (μmol/L)	Precalving	322.13±49.80^a^	347.96±64.95^a^	409.32±58.72^a^	571.37±68.07^b^	<0.01	0.61	0.97
	Calving	345.75±34.87^a^	324.50±52.98^a^	345.83±60.62^a^	549.92±89.82^b^			
	Postcalving	369.01±50.22^a^	343.23±47.27^a^	388.38±83.62^a^	559.71±66.13^b^			

BCS, body condition score.

1) MBCS, medium BCS (3.0 to 3.25); HBCS, high BCS (3.5 to 3.75); HerBCS, higher BCS (4.0 to 4.25); HestBCS, highest BCS (4.5 to 5.0).

a,b Means in the same row followed by different superscripts differ at p<0.05.

A,B Means in the same column followed by different superscripts differ at p<0.05.

**Table 4:** Influence of body condition score on oxidative status of Holstein cows

Variables		Treatments1)				p-value		
	
		MBCS (3.0 to 3.25)	HBCS (3.5 to 3.75)	HerBCS (4.0 to 4.25)	HestBCS (4.5 to 5.0)	BCS	Time	BCS×time
MDA (nmol/mL)	Precalving	8.75±2.37^a^	8.84±1.49^a^^AB^	9.05±1.28^a^^AB^	12.64±1.81^b^^B^	<0.01	<0.05	0.16
	Calving	9.13±1.19^a^	9.48±1.16^a^^B^	9.54±2.1^a^^B^	17.17±2.42^b^^C^	<0.01		
	Postcalving	8.59±1.97^ab^	6.93±1.96^a^^A^	6.69±1.52^a^^A^	10.03±1.23^b^^A^	<0.01		
SOD (U/mL)	Precalving	177.40±27.52^a^	171.08±22.67^a^^B^	180.14±18.03^a^^B^	256.79±25.84^b^^A^	<0.01	<0.05	0.05
	Calving	191.46±32.91^a^	177.01±23.70^a^^B^	192.71±25.65^a^^B^	348.58±23.43^b^^B^	<0.01		
	Postcalving	183.98±24.94^b^	125.09±19.07^a^^A^	131.74±20.74^a^^A^	216.57±26.10^c^^A^	<0.01		
CAT (U/mL)	Precalving	139.96±27.46^a^	137.81±28.03^a^	132.66±18.75^a^	196.25±32.81^b^	<0.01	0.51	0.47
	Calving	126.49±18.16^a^	120.44±18.30^a^	119.56±13.79^a^	198.61±20.01^b^	<0.01		
	Postcalving	148.05±27.20^a^	122.45±45.15^a^	144.79±59.91^a^	207.02±45.58^b^	<0.01		
VA (ng/mL)	Precalving	79.45±15.02^a^^B^	61.36±20.36^a^^A^	62.24±21.59^a^^A^	113.15±25.84^b^^A^	<0.01	<0.05	0.05
	Calving	65.60±11.42^a^^B^	64.89±13.09^a^^A^	71.44±13.44^a^^A^	123.20±19.61^b^^AB^			
	Postcalving	62.22±12.64^a^^A^	108.88±11.37^b^^B^	123.63±24.73^b^^B^	135.30±9.55^c^^AB^			
VE (ug/mL)	Precalving	35.24±5.99^B^	29.54±6.51	29.12±6.43	36.90±9.51	<0.01	0.13	0.37
	Calving	33.55±5.79^a^^B^	32.15±2.34^a^	36.17±9.96^a^	46.70±9.31^b^			
	Postcalving	26.09±5.21^a^^A^	30.14±8.91^a^	31.93±8.32^ab^	40.62±9.39^b^			

BCS, body condition score; MDA, malondialdehyde; SOD, oxidase dismutase; CAT, catalase; VA, vitamin A; VE, vitamin E.

1) MBCS, medium BCS (3.0 to 3.25); HBCS, high BCS (3.5 to 3.75); HerBCS, higher BCS (4.0 to 4.25); HestBCS, highest BCS (4.5 to 5.0).

a,b Means in the same row followed by different superscripts differ at p<0.05.

A,B Means in the same column followed by different superscripts differ at p<0.05.

**Table 5:** Pearson correlation coefficients of the levels of non-esterified fatty acid and beta-hydroxybutyrate, and indices of oxidative status and body condition score of Holstein cows

	NEFA	BHBA	MDA	SOD	CAT	VA	VE	BCS
NEFA	-							
BHBA	0.572**	-						
MDA	0.468**	0.764**	-					
SOD	0.379**	0.624**	0.781**	-				
CAT	0.524**	0.750**	0.585**	0.594**	-			
VA	0.354**	0.636**	0.375**	0.487**	0.512**	-		
VE	0.417**	0.788**	0.741**	0.671**	0.527**	0.639**	-	
BCS	0.260*	0.496**	0.700**	0.606**	0.342**	0.245*	0.448**	-

NEFA, non-esterified fatty acid; BHBA, beta-hydroxybutyrate; MDA, malondialdehyde; SOD, oxidase dismutase; CAT, catalase; VA, vitamin A; VE, vitamin E; BCS: body condition score.

* p<0.05,

** p<0.01.
